# Personalized mRNA Neoantigen Vaccines in Cancer: Linking Precision Antigen Selection, Immune Remodeling, and Checkpoint Blockade

**DOI:** 10.3390/ijms27188163

**Published:** 2026-09-14

**Authors:** Turan Demircan, Berna Yıldırım, Halil İbrahim Ünsal, Ayhan Bilir

**Affiliations:** 1 Medical Biology Department, School of Medicine, İzmir Bakırçay University, İzmir 35665, Türkiye; turandemircan@gmail.com; 2 Department of Histology and Embryology, Faculty of Medicine, Istanbul Atlas University, Istanbul 34403, Türkiye; berna.yildirim@atlas.edu.tr; 3 Department of Family Medicine, Provincial Directorate of Health, Diyarbakir 21120, Türkiye; halilibrahimunsal@hotmail.com

**Keywords:** personalized mRNA neoantigen vaccine, cancer vaccine, neoantigen, immune checkpoint blockade, immune remodeling, tumor microenvironment, antigen presentation, tumor heterogeneity, melanoma, precision immunotherapy

## Abstract

Personalized mRNA neoantigen vaccines represent an emerging form of precision cancer immunotherapy designed to generate de novo T-cell responses against patient-specific tumor mutations. Their therapeutic activity, however, depends not only on neoantigen selection and vaccine-induced immune priming but also on whether these responses can remain functional within an immunosuppressive tumor microenvironment. Immune checkpoint blockade provides a mechanistically complementary strategy by relieving inhibitory signaling that constrains vaccine-expanded tumor-reactive lymphocytes. This review examines this therapeutic interface across three interconnected levels: molecular neoantigen selection and mRNA vaccine design, vaccine-driven remodeling of antitumor T-cell immunity, and clinical integration with PD-1/PD-L1 blockade. Particular emphasis is placed on how neoantigen clonality, antigen presentation, tumor heterogeneity, and immune escape influence the translation of vaccine immunogenicity into clinical benefit. Current evidence is evaluated across melanoma, non-small cell lung cancer, and pancreatic ductal adenocarcinoma, including randomized and ongoing trials of intismeran autogene, an individualized synthetic mRNA therapy encoding patient-specific neoantigens, and autogene cevumeran, an individualized uridine mRNA-lipoplex neoantigen vaccine. By integrating molecular vaccine design with tumor evolution, immune-contexture remodeling, and checkpoint sensitivity, this review identifies determinants that may distinguish successful immune priming from durable therapeutic efficacy and outlines priorities for biomarker-guided patient selection and future combination strategies.

## 1. Introduction

Cancer immunotherapy has been transformed by the clinical success of immune checkpoint inhibitors (ICIs) targeting programmed cell death protein 1 (PD-1), programmed death-ligand 1 (PD-L1), and cytotoxic T-lymphocyte-associated protein 4 (CTLA-4) [1,2]. By disrupting inhibitory signaling pathways that constrain antitumor immunity, ICIs can restore or enhance tumor-reactive T-cell function and produce durable clinical responses in a subset of patients. Nevertheless, primary and acquired resistance remain major limitations across solid tumors, including melanoma and non-small cell lung cancer (NSCLC) [1,2]. Resistance to immune checkpoint blockade is multifactorial and may involve insufficient T-cell priming, defective antigen processing and presentation, T-cell exclusion, loss of tumor antigenicity, and the establishment of an immunosuppressive tumor microenvironment [1,2]. Consequently, tumors with limited pre-existing tumor-reactive T-cell immunity, often described as immunologically “cold”, may derive comparatively little benefit from checkpoint blockade alone [1,2]. These limitations provide a strong rationale for therapeutic strategies that actively generate, broaden, and sustain tumor-specific T-cell immunity rather than relying exclusively on the reinvigoration of pre-existing immune responses [3,4].

Personalized cancer vaccines targeting tumor neoantigens represent one such strategy. Neoantigens arise predominantly from nonsynonymous somatic alterations and can generate peptide sequences that are absent from normal tissues [3]. Because these mutation-derived epitopes are less constrained by central immune tolerance than shared self-antigens, they provide attractive targets for highly tumor-specific T-cell responses [3]. Among the available neoantigen-delivery platforms, messenger RNA (mRNA) vaccines are particularly adaptable to individualized cancer immunotherapy [5]. Sequence-based manufacturing enables patient-specific vaccine constructs to be generated from tumor genomic and transcriptomic information, while a single mRNA construct can encode multiple selected neoepitopes. Advances in lipid nanoparticle (LNP)- and related RNA-delivery technologies further facilitate intracellular antigen expression and support increasingly flexible vaccine-production strategies [5]. Importantly, however, the therapeutic potential of an individualized mRNA vaccine is determined not only by the identification and encoding of immunogenic neoantigens, but also by the subsequent biological context in which vaccine-induced T cells are primed, expanded, trafficked to tumors, and exposed to mechanisms of immune suppression.

Personalized mRNA neoantigen vaccination and immune checkpoint blockade (ICB) therefore act at complementary stages of the antitumor immune response. Vaccination can induce de novo priming and increase the magnitude and breadth of neoantigen-specific T-cell populations, whereas checkpoint blockade can preserve or restore the function of these vaccine-expanded lymphocytes after they encounter inhibitory signaling within the tumor microenvironment [4,6]. Experimental evidence supports this mechanistic interaction, demonstrating that concurrent checkpoint blockade can modify the expansion, persistence, and functional dynamics of neoantigen vaccine-induced antitumor T-cell populations [4,6]. The clinical translation of this concept is currently most advanced with intismeran autogene, formerly designated mRNA-4157/V940, in combination with pembrolizumab. In the randomized phase IIb KEYNOTE-942 study involving patients with completely resected high-risk melanoma, intismeran autogene plus pembrolizumab improved recurrence-free and distant metastasis-free survival compared with pembrolizumab alone, with the treatment effect remaining evident after approximately five years of follow-up [7,8]. The same individualized mRNA platform is undergoing phase III evaluation in NSCLC, including postoperative settings in which vaccination is being investigated as a strategy to broaden antitumor immunity against residual or micrometastatic disease [9,10]. In pancreatic ductal adenocarcinoma (PDAC), individualized RNA neoantigen vaccination has additionally provided evidence that de novo vaccine-induced T-cell populations can remain detectable and functionally active for several years even in a tumor type characterized by low baseline immunogenicity [11].

The central focus of this review is therefore not the mRNA vaccine platform or immune checkpoint blockade in isolation, but the molecular and translational interface that determines whether personalized neoantigen vaccination can generate clinically meaningful antitumor immunity. We organize this interface across three interconnected levels. First, we examine precision neoantigen selection and individualized mRNA vaccine design, including tumor sequencing, antigen prioritization, construct generation, and delivery. Second, we analyze how vaccine-induced T-cell priming and expansion interact with antigen presentation, tumor heterogeneity, immune escape, and checkpoint-mediated suppression within the tumor microenvironment. Third, we evaluate how these biological determinants are reflected in current clinical development across melanoma, NSCLC, and PDAC, which represent distinct stages of translational maturity: melanoma currently provides the strongest randomized efficacy signal, NSCLC represents an advanced phase III development setting, and PDAC provides evidence of durable vaccine-induced cellular immunity in an immunologically challenging tumor type [7,8,9,10,11]. By linking precision antigen selection with immune-contexture remodeling, tumor evolution, and checkpoint sensitivity, this review aims to identify the biological determinants that may distinguish successful vaccine immunogenicity from durable therapeutic efficacy. We further examine manufacturing feasibility, accessibility, biomarker development, and patient-selection strategies that will influence the broader clinical implementation of personalized mRNA neoantigen vaccines.

## 2. Mechanistic Rationale

### 2.1. Personalized mRNA Vaccine Workflow

Personalized mRNA neoantigen vaccine development begins with integrated molecular profiling of matched tumor and normal tissue to identify tumor-specific alterations and determine whether the corresponding mutant alleles are expressed. Candidate neoantigens are subsequently prioritized using genomic, transcriptomic, HLA-binding, clonality, and increasingly immunopeptidomic information. Proteogenomic approaches can provide direct evidence that predicted tumor-derived peptides are naturally presented by MHC molecules, thereby reducing reliance on sequence-based prediction alone [3,12]. Accordingly, a computationally predicted neoantigen should be distinguished from a naturally presented and experimentally immunogenic neoantigen. Sequence-based prediction identifies candidates with favorable molecular characteristics, whereas mass spectrometry-based immunopeptidomics provides direct evidence of peptide-HLA presentation, and functional T-cell assays are required to demonstrate immune recognition. These levels of evidence are not interchangeable, and increasingly stringent validation may improve the biological relevance of vaccine target selection [3,12,13,14].

Neoantigen prioritization: Candidate neoepitopes are evaluated according to multiple features that influence their probability of generating a productive T-cell response. These include predicted binding to the patient’s HLA class I and class II molecules, mutant-allele expression, peptide processing and presentation, clonality, and the distinction between mutant and corresponding wild-type sequences [3]. Contemporary prediction pipelines increasingly incorporate machine-learning algorithms trained on experimentally characterized HLA ligandomes and other immunopeptidomic datasets [12,13,14]. Because tumor sequencing may identify substantially more candidate alterations than can be incorporated into an individualized vaccine, computational frameworks are required to rank and curate the most promising targets. Tools such as pVACview facilitate systematic neoantigen prioritization by integrating candidate information across variant, transcript, peptide, and prediction-algorithm levels within an interactive interface. Rather than relying on a single ranking metric, pVACview allows investigators to inspect and compare features such as transcript annotations, peptide candidates, predicted HLA-binding and presentation characteristics, and other candidate-level attributes, and subsequently select and export prioritized neoantigens for downstream vaccine design [13].

Tumor mutational burden (TMB) provides a broad estimate of the mutational substrate from which candidate neoantigens may arise, but mutation quantity alone does not reliably predict neoantigen quality or immunogenicity. High-TMB tumors may contain many poorly presented or immunologically irrelevant mutations, whereas low-TMB tumors can harbor a limited number of highly immunogenic and therapeutically actionable neoantigens [15]. Accordingly, current strategies are moving beyond mutation counts toward integrated prioritization frameworks incorporating expression, clonality, HLA presentation, structural properties, immunopeptidomic evidence, and increasingly artificial intelligence-assisted prediction of neoantigen immunogenicity [14]. This transition from mutation enumeration to multidimensional target selection is central to improving the precision of individualized vaccine design.

Shared and individualized neoantigen strategies differ primarily in how recurrent oncogenic mutations are incorporated into vaccine design. Shared or “off-the-shelf” approaches target recurrent driver mutations that occur across multiple patients, provided that the corresponding mutant peptide can be presented by an appropriate HLA allele. Frequently investigated examples include hotspot mutations in KRAS and TP53. In contrast, fully individualized vaccines derive their antigen composition from the unique mutational landscape of each patient and may therefore incorporate both recurrent driver mutations and private passenger mutations, provided that these candidates are expressed, clonally represented, and predicted or experimentally demonstrated to generate suitable peptide-HLA targets. This distinction is clinically important because recurrent driver mutations offer broader population applicability, whereas individualized multiepitope designs can capture a wider spectrum of tumor-specific antigens and potentially reduce vulnerability to escape through loss of a single target.

A clinically informative example is provided by the SLATE platform, which used a heterologous chimpanzee adenovirus/self-amplifying mRNA vaccine encoding 20 shared neoantigens derived from recurrent oncogenic driver mutations. In the phase I component of NCT03953235, 18 of 19 treated patients harbored KRAS-mutant tumors, while the vaccine also contained HLA-matched TP53-derived neoepitopes. Interestingly, vaccine-induced T-cell responses were disproportionately directed toward TP53 neoantigens rather than the KRAS mutations expressed by most tumors, revealing an immunodominance hierarchy that may limit the effectiveness of multiepitope shared vaccines. This observation prompted subsequent optimization toward a KRAS-focused antigen set and illustrates that inclusion of a driver mutation in a vaccine does not by itself guarantee dominant or therapeutically relevant T-cell recognition [16]. By comparison, clinically advanced individualized mRNA platforms construct a distinct antigen repertoire for each patient: intismeran autogene can encode up to 34 patient-specific neoantigens, whereas autogene cevumeran has been designed to encode up to 20 selected patient-specific neoantigens [7,17,18].

**Figure 1:**
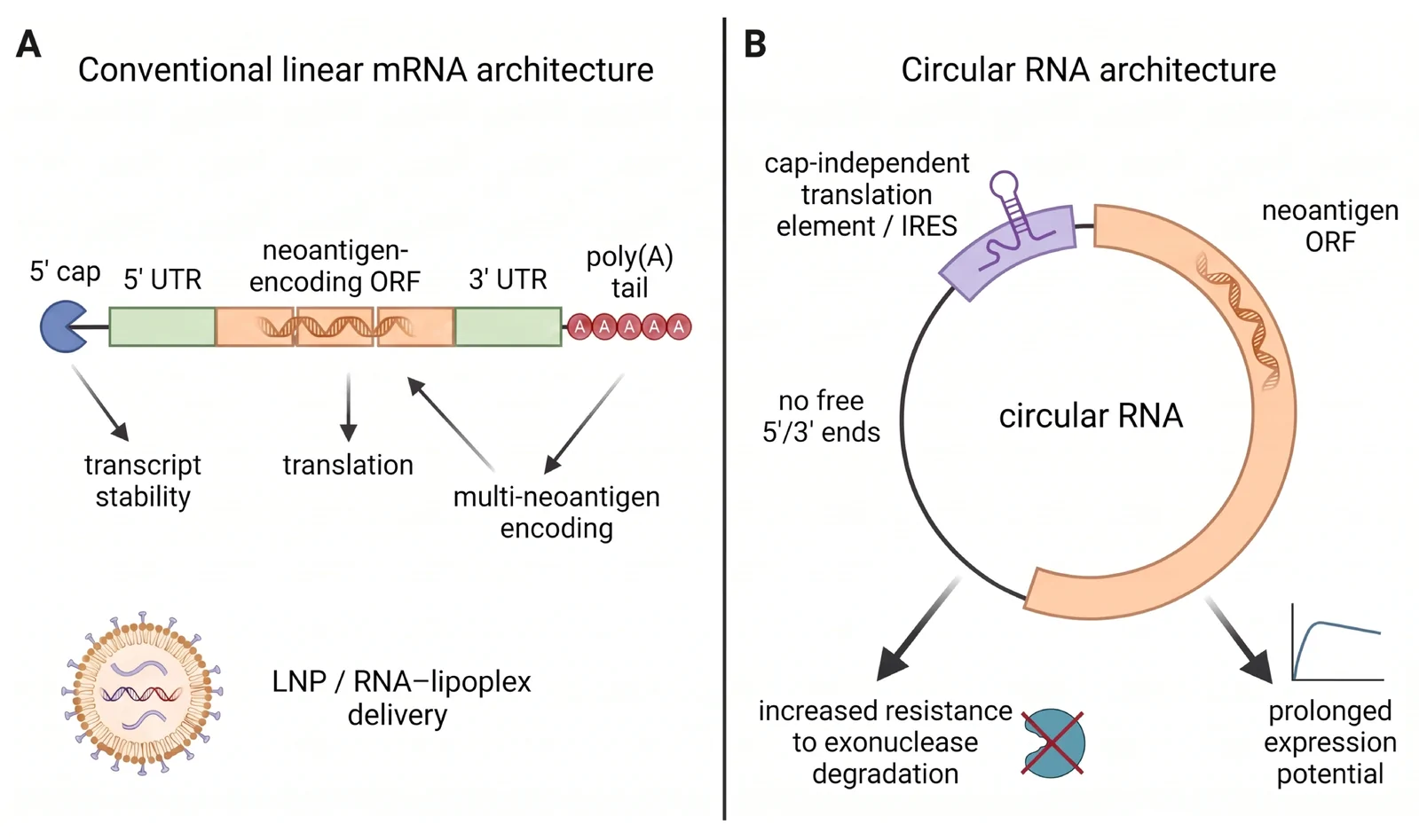
Conventional and alternative RNA architectures relevant to personalized neoantigen vaccination. (**A**) Conventional linear mRNA contains a 5′ cap, 5′ untranslated region (UTR), neoantigen-encoding open reading frame (ORF), 3′ UTR, and poly(A) tail. These structural elements support transcript stability and efficient translation, while multiple neoantigen sequences can be incorporated within a single construct. Delivery is typically mediated by lipid-based formulations, including lipid nanoparticles (LNPs) or RNA-lipoplex systems. (**B**) Circular RNA (circRNA) lacks free 5′ and 3′ ends and can support cap-independent translation through appropriate regulatory elements such as an internal ribosome entry site (IRES). Its covalently closed structure may increase resistance to exonuclease-mediated degradation and prolong antigen expression. Circular RNA neoantigen vaccines remain predominantly at the preclinical stage [5,19]. (Created in BioRender.com; Science Suite Inc. dba BioRender, Toronto, ON, Canada).

mRNA construct generation and delivery: Following neoantigen prioritization, multiple patient-specific targets can be encoded within a single RNA construct, enabling simultaneous targeting of distinct tumor clones and reducing dependence on any single antigen. Platform-specific differences in RNA architecture and delivery, including conventional linear mRNA, emerging circular RNA formats, and delivery through lipid nanoparticle or RNA-lipoplex formulations, primarily influence transcript stability, biodistribution, antigen expression, and cellular targeting [5,17,19]. The canonical linear mRNA structure and representative alternative RNA architectures, including circular RNA, are summarized schematically in Figure 1 [5,19]. Rather than reviewing these established principles in detail, we focus here on those design variables that directly affect personalized neoantigen selection and clinical translation.

Route of administration and treatment scheduling constitute additional determinants of vaccine biodistribution and immune priming. In the clinically advanced personalized neoantigen platforms discussed here, intismeran autogene is administered intramuscularly, whereas autogene cevumeran is delivered intravenously as an RNA-lipoplex formulation [7,18].

Vaccination and clinical feasibility: A central practical requirement of personalized vaccination is completion of the entire workflow within a clinically actionable time frame. This has been demonstrated in resected pancreatic ductal adenocarcinoma (PDAC), in which individualized RNA neoantigen vaccines were generated from surgically obtained tumor material and integrated into a postoperative treatment regimen. In the phase I study of autogene cevumeran, vaccination induced de novo, high-magnitude neoantigen-specific T-cell responses in 8 of 16 evaluable patients, demonstrating that individualized mRNA vaccine production and administration can be incorporated into a complex perioperative oncology workflow [18]. Extended follow-up of this cohort further demonstrated that vaccine-induced CD8^+^ T-cell clones can persist for years and remain functionally detectable, while patients who developed vaccine-induced T-cell responses showed prolonged recurrence-free survival compared with non-responders. Because this was a small, non-randomized study, the association between immune response and recurrence cannot establish clinical efficacy; nevertheless, the findings provide important proof of concept that individualized mRNA vaccination can generate durable and biologically meaningful antitumor immunity [11]. Further optimization of the workflow is therefore focused on improving neoantigen identification and proteogenomic validation, enhancing candidate selection in tumors with relatively low mutational burdens, and shortening the interval between tissue acquisition and vaccine administration [12,20]. The feasibility of identifying personalized vaccine targets in low-TMB NSCLC further illustrates that potentially actionable neoantigens may be recoverable even from tumors with relatively restricted mutational landscapes, although such observations should not be interpreted as evidence of clinical efficacy for mRNA vaccination in this setting [21].

### 2.2. Vaccine-Induced T-Cell Priming, Tumor-Mediated Immune Suppression, and Checkpoint Modulation

Antigen presentation and de novo T-cell priming: The principal translational objective of personalized vaccination is not simply antigen expression but the generation of sufficiently broad, high-avidity CD4^+^ and CD8^+^ T-cell populations against patient-specific neoantigens. Because mutation-derived epitopes are absent from normal tissues and less constrained by central tolerance, vaccination can recruit previously unprimed tumor-reactive clones and may additionally promote epitope spreading beyond the initially encoded targets [3,4,22].

Tumor-mediated immunosuppression as a limiting step: Successful peripheral priming does not necessarily translate into effective tumor control because vaccine-expanded T cells subsequently encounter multiple inhibitory mechanisms within the tumor microenvironment. Persistent antigen exposure and chronic inflammatory signaling can promote sustained expression of inhibitory receptors, including PD-1, and contribute to progressive T-cell dysfunction characterized by impaired proliferative capacity, reduced cytokine production, and diminished cytotoxic activity [6]. Tumors can additionally evade vaccine-induced immunity through quantitative or qualitative defects in antigen presentation, including reduced expression of HLA molecules or components of the antigen-processing machinery, thereby limiting recognition by otherwise competent tumor-reactive T cells [23]. Intratumoral heterogeneity introduces a further layer of resistance: neoantigens may be expressed clonally across the tumor population or restricted to subclonal compartments, allowing antigen-negative or poorly recognized tumor populations to persist despite effective immune pressure against vaccine-targeted clones [24]. Because direct presentation of the targeted epitope by malignant cells is required for recognition and killing by cognate CD8^+^ T cells, loss of neoantigen expression or antigen-presentation competence can undermine clinical activity even when vaccination generates robust systemic immune responses [23]. Immune selection can further reshape this landscape over time through loss of targeted neoantigens, HLA alleles, or components of the antigen-processing machinery, thereby converting an initially relevant vaccine target into an immunologically inaccessible one [23,24].

Checkpoint modulation of vaccine-induced immunity: Personalized neoantigen vaccination and immune checkpoint blockade act at complementary stages of the cancer-immunity cycle rather than functioning as redundant immunotherapeutic interventions [4,25]. Vaccination expands the pool and diversity of tumor-reactive T cells, whereas checkpoint blockade can preserve or restore the function of these lymphocytes after they encounter inhibitory signaling within the tumor microenvironment [4,25]. Preclinical studies have shown that concurrent checkpoint blockade can alter the dynamics of vaccine-induced T-cell populations, sustaining their expansion and functional competence and improving antitumor activity relative to either intervention alone in appropriate experimental settings [4]. Consistent with this model, mRNA vaccination has been shown in immunologically cold tumor models to promote a more T-cell-inflamed microenvironment characterized by enhanced recruitment of cytotoxic lymphocytes and increased expression of inflammatory chemokines, thereby creating conditions that may favor improved responsiveness to PD-1/PD-L1 blockade [25].

**Figure 2:**
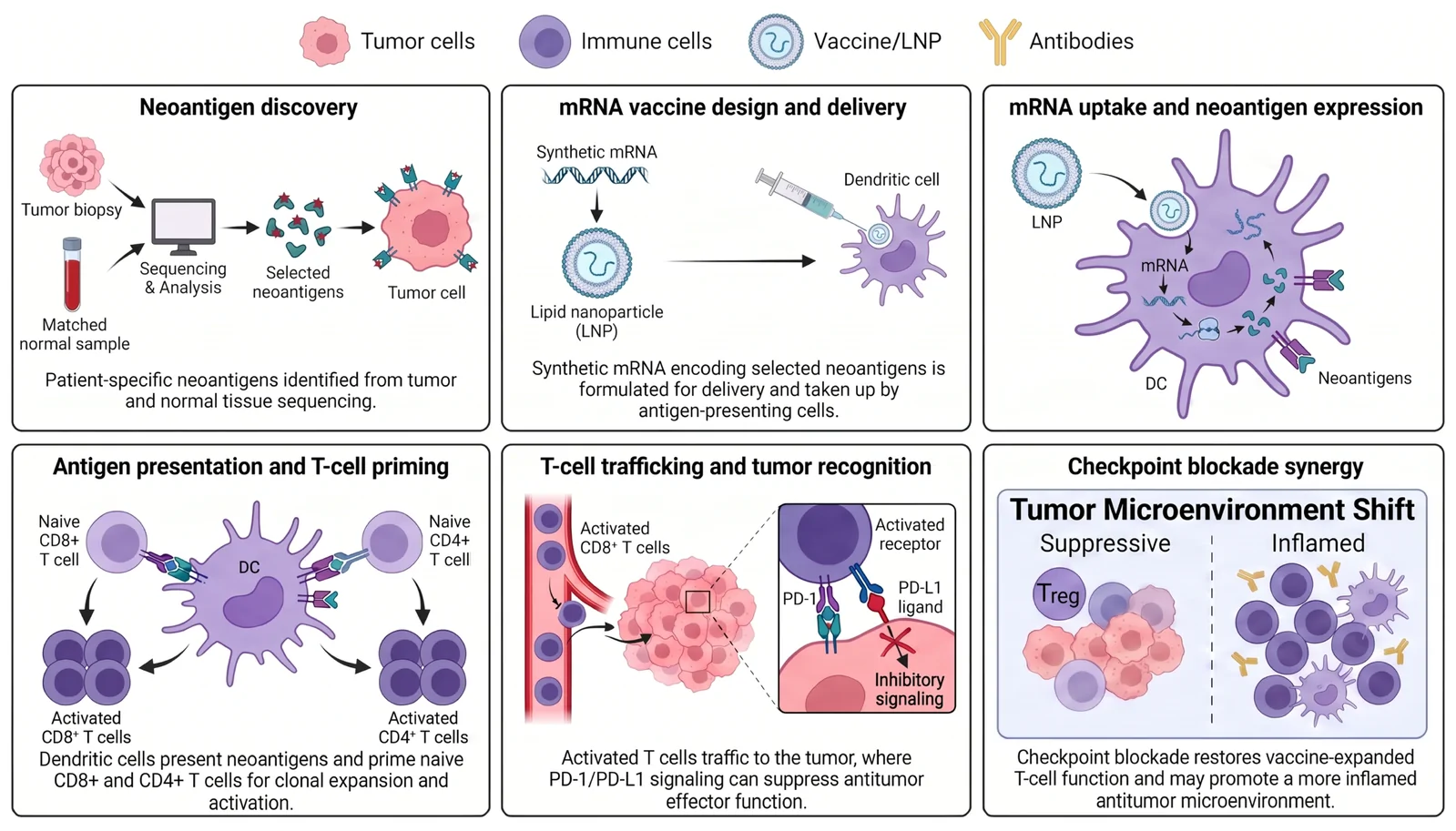
Molecular and immunological framework of personalized mRNA neoantigen vaccination and its interaction with PD-1/PD-L1 checkpoint blockade. Patient-specific neoantigens are identified through sequencing and bioinformatic analysis of matched tumor and normal samples. Selected neoantigens are then encoded into a synthetic mRNA construct, formulated for delivery, and taken up by antigen-presenting cells such as dendritic cells (DCs). Following intracellular release and translation, vaccine-derived neoantigens are processed and presented through major histocompatibility complex (MHC) class I and class II pathways, promoting priming and clonal expansion of naïve CD8^+^ and CD4^+^ T cells. Activated T cells subsequently traffic to the tumor microenvironment, where recognition of tumor cells may be limited by inhibitory PD-1/PD-L1 signaling. Immune checkpoint blockade can restore the effector function of vaccine-expanded T cells, enhance tumor-cell killing, and may promote a shift toward a more inflamed antitumor microenvironment [3,4,23,25] (Created in BioRender.com; Science Suite Inc. dba BioRender, Toronto, ON, Canada).

Importantly, these findings support a biologically complementary interaction between vaccination and checkpoint inhibition, but they do not establish that clinically meaningful synergy will occur uniformly across tumor types or treatment settings. The magnitude of benefit is likely to depend on factors including antigenic architecture, neoantigen clonality, antigen-presentation competence, baseline immune infiltration, and the degree of pre-existing immune suppression [24,25]. The interaction between personalized mRNA neoantigen vaccination and PD-1/PD-L1 blockade is summarized schematically in Figure 2.

Emerging translational observations in melanoma further support the possibility that mRNA vaccination can modify the composition and functional state of the tumor immune microenvironment in ways that may enhance responsiveness to checkpoint inhibition [26]. However, such correlative evidence should not be interpreted as definitive proof that the clinical benefit of combination therapy reflects intrinsic biological synergy in all treatment settings. Rather, the available evidence supports a mechanistic framework in which vaccination increases the availability, breadth, and diversity of tumor-reactive lymphocytes, while checkpoint inhibition reduces inhibitory constraints on their function.

Related principles have also been demonstrated using alternative RNA vaccine architectures. For example, a personalized circular RNA neoantigen vaccine generated potent antitumor immune responses in melanoma models and achieved greater tumor control when combined with checkpoint blockade than either intervention alone [19]. Combination strategies involving other vaccine platforms have similarly been explored. In dendritic cell-based cancer vaccination, checkpoint inhibition has been proposed as a means of overcoming the limited persistence and functional activity of vaccine-induced T-cell populations within immunosuppressive tumors [22]. Together, these observations suggest that the biological value of checkpoint inhibition may extend beyond direct reinvigoration of pre-existing antitumor immunity by also preserving or enhancing newly generated vaccine-induced immune responses.

Effects on the broader tumor immune contexture: Beyond effects on individual neoantigen-specific clones, vaccination may promote CD8^+^ T-cell infiltration and inflammatory chemokine expression, potentially shifting tumors toward a more checkpoint-responsive immune contexture [25]. However, this remodeling remains constrained by suppressive myeloid populations, regulatory T cells, defective antigen presentation, and heterogeneous neoantigen expression [23,24,25]. Thus, systemic vaccine immunogenicity should not be equated with tumor control; therapeutic benefit ultimately requires vaccine-expanded lymphocytes to access, recognize, and remain functional against evolving malignant-cell populations.

## 3. Clinical Progress

### 3.1. Melanoma: The Most Mature Clinical Experience

Melanoma currently provides the most mature clinical evidence for combining personalized mRNA neoantigen vaccination with immune checkpoint blockade. This setting is biologically favorable because melanoma frequently exhibits a relatively high tumor mutational burden and substantial neoantigen diversity, together with an immunologically active tumor microenvironment and established sensitivity to PD-1 inhibition [27,28]. The adjuvant setting may be particularly suitable for individualized vaccination because complete surgical resection reduces macroscopic tumor burden and may diminish tumor-mediated immunosuppression while allowing sufficient time for vaccine-induced neoantigen-specific T-cell responses to develop [7,28]. Within this context, intismeran autogene, formerly designated mRNA-4157/V940, combined with pembrolizumab has emerged as the most clinically advanced individualized mRNA neoantigen vaccine strategy in melanoma [27,28].

Early clinical evidence for this platform was provided by the first-in-human phase I KEYNOTE-603 study (NCT03313778) [29]. Intismeran autogene induced de novo and enhanced pre-existing neoantigen-specific T-cell responses in patients with resected NSCLC, while combination treatment with pembrolizumab in patients with resected cutaneous melanoma generated sustained vaccine-induced responses involving both CD8^+^ and CD4^+^ T-cell populations. No grade 4 or 5 treatment-related adverse events or dose-limiting toxicities were reported in the analyzed cohorts [29]. These findings established proof of concept that individualized mRNA vaccination could generate measurable and persistent neoantigen-specific immunity in patients and provided a biological basis for subsequent randomized development with PD-1 blockade.

Intismeran autogene is an individualized lipid nanoparticle-formulated mRNA therapy designed on the basis of each patient’s tumor mutational profile and capable of encoding up to 34 patient-specific neoantigens. Its clinical activity in melanoma was evaluated in the randomized, open-label phase IIb KEYNOTE-942/mRNA-4157-P201 study (NCT03897881), which enrolled 157 patients with completely resected, high-risk stage IIIB–IV cutaneous melanoma [7,29]. Patients were randomly assigned in a 2:1 ratio to receive nine intramuscular doses of intismeran autogene plus adjuvant pembrolizumab or pembrolizumab alone. In the primary analysis, recurrence or death occurred in 22% of patients in the combination group and 40% of those receiving pembrolizumab alone. The combination prolonged recurrence-free survival (RFS), with a hazard ratio (HR) of 0.561 (95% CI, 0.309–1.017; two-sided *p* = 0.053), while the estimated 18-month RFS rates were 79% and 62%, respectively. Although the conventional two-sided *p* value was 0.053, the primary endpoint met the study’s prespecified statistical criterion. The combination also improved distant metastasis-free survival (DMFS), with an HR of 0.347 (95% CI, 0.145–0.828) [7]. These findings provided the first randomized clinical evidence that adding an individualized mRNA neoantigen vaccine to established PD-1 blockade could improve clinically relevant disease-control endpoints.

Extended follow-up has strengthened the evidence that this treatment effect is durable. At approximately three years of follow-up, intismeran autogene plus pembrolizumab maintained an RFS advantage over pembrolizumab alone, with an HR of 0.510 (95% CI, 0.288–0.906), while the HR for DMFS was 0.384, corresponding to relative reductions of approximately 49% in the risk of recurrence or death and 62% in the risk of distant metastasis or death [30]. More recently, the planned five-year analysis of KEYNOTE-942 demonstrated continued separation of the treatment groups after a median planned follow-up of 60.3 months. The RFS HR remained 0.510 (95% CI, 0.294–0.887), while the DMFS HR was 0.411 (95% CI, 0.200–0.843). Overall survival (OS) showed a favorable trend for the combination, with an HR of 0.471 (95% CI, 0.165–1.345), although the low number of deaths and wide confidence interval preclude definitive conclusions regarding an OS benefit [8]. No new safety signals emerged during extended follow-up [8].

The translational analyses accompanying the five-year follow-up are particularly relevant to the proposed mechanism of personalized vaccination. Compared with pembrolizumab alone, intismeran autogene plus pembrolizumab was associated with increased T-cell receptor clonality and a greater magnitude of newly emerging T-cell clonotypes. Within the combination arm, greater expansion of novel clones was observed in patients who remained recurrence-free than in those who subsequently experienced recurrence [8]. These findings are consistent with the hypothesis that individualized neoantigen vaccination can expand and diversify the antitumor T-cell repertoire beyond that generated by PD-1 blockade alone. Importantly, however, these translational associations remain correlative and do not establish a direct causal relationship between repertoire remodeling and clinical benefit.

Development of intismeran autogene has also expanded beyond the resected setting. INTerpath-012 (NCT06961006) is an ongoing randomized phase II study evaluating first-line intismeran autogene plus pembrolizumab versus placebo plus pembrolizumab in patients with unresectable advanced melanoma [31]. This trial will help determine whether the biological and clinical advantages observed in the minimal-residual-disease setting can be reproduced in patients with established macroscopic disease, where tumor burden, heterogeneity, and immunosuppression are substantially greater.

The phase III INTerpath-001/V940-001 trial (NCT05933577) has now provided the first confirmatory phase III efficacy signal for an individualized mRNA neoantigen therapy. In this randomized, double-blind, placebo- and active-comparator-controlled study, patients with completely resected stage IIB–IV cutaneous melanoma received adjuvant intismeran autogene plus pembrolizumab or placebo plus pembrolizumab [32]. At a prespecified interim analysis announced in August 2026, the combination met the primary endpoint of recurrence-free survival and the key secondary endpoint of distant metastasis-free survival, with statistically significant and clinically meaningful improvements over pembrolizumab alone [33]. The safety profile was reported to be consistent with previous experience, with no new safety signals identified [33]. Detailed effect estimates and mature overall-survival data have not yet been publicly reported; therefore, interpretation should remain limited to the positive topline efficacy announcement pending full presentation and peer-reviewed publication. Nevertheless, INTerpath-001 represents the first positive phase III readout for an individualized neoantigen therapy and substantially strengthens the clinical evidence supporting personalized mRNA vaccination in the adjuvant melanoma setting [33].

### 3.2. Non-Small Cell Lung Cancer: Testing Personalized mRNA Vaccination in High-Risk Perioperative Disease

Following the encouraging clinical activity observed in resected melanoma, non-small cell lung cancer (NSCLC) has emerged as an important test of whether personalized mRNA neoantigen vaccination can improve outcomes in a biologically and immunologically more heterogeneous malignancy. This extension is clinically relevant because immune checkpoint inhibitors are now incorporated into both adjuvant and perioperative NSCLC treatment strategies, yet a substantial proportion of patients still experience recurrence after curative-intent resection [34,35]. NSCLC encompasses molecularly and immunologically distinct subgroups: smoking-associated tumors frequently harbor relatively high mutational and neoantigen burdens, whereas oncogene-driven tumors and cancers arising in never-smokers often exhibit lower tumor mutational burden (TMB), reduced immune infiltration, and less favorable responses to immune checkpoint inhibition [15,34,35]. Accordingly, the NSCLC development program represents not only a test of the clinical activity of intismeran autogene, but also a test of whether individualized vaccination can adapt to a heterogeneous and evolutionarily dynamic neoantigen landscape.

Intismeran autogene combined with pembrolizumab is currently being evaluated in two complementary randomized phase III trials in resectable NSCLC. INTerpath-002 (NCT06077760) is a randomized, double-blind, placebo- and active-comparator-controlled study evaluating adjuvant intismeran autogene plus pembrolizumab versus placebo plus pembrolizumab in an estimated 868 patients with completely resected, margin-negative stage II, IIIA, or IIIB (N2) NSCLC who have not received neoadjuvant systemic therapy. Disease-free survival (DFS) is the primary endpoint [10]. INTerpath-009 (NCT06623422) addresses a distinct high-risk population and evaluates adjuvant pembrolizumab with or without intismeran autogene in an estimated 680 patients with resectable stage II–IIIB (N2) NSCLC who fail to achieve a pathological complete response after neoadjuvant pembrolizumab plus platinum-based doublet chemotherapy and subsequent surgery [9]. DFS is also the primary endpoint of this study. As of August 2026, neither trial has reported efficacy results [9,10]. The clinical development program has additionally expanded to metastatic disease through INTerpath-013 (NCT07221474), an ongoing randomized phase II study evaluating intismeran autogene or placebo in combination with pembrolizumab and platinum-based chemotherapy as first-line treatment for metastatic squamous NSCLC [36].

The residual-disease design of INTerpath-009 is particularly informative from a translational perspective. Failure to achieve a pathological complete response indicates persistence of viable tumor despite neoadjuvant chemoimmunotherapy and identifies a population that remains at substantial risk of postoperative recurrence. In this setting, an individualized neoantigen vaccine could theoretically broaden the repertoire of tumor-reactive T cells directed against residual malignant clones, whereas continued PD-1 blockade could preserve or restore the function of vaccine-expanded lymphocytes within the postoperative micrometastatic compartment. This strategy can therefore be conceptualized as a form of immune consolidation after incomplete pathological response [9,34]. Importantly, however, this rationale remains mechanistic and hypothesis-generating until the trial demonstrates that vaccine-induced immunity translates into a clinically meaningful improvement in DFS, overall survival, or other prespecified outcomes.

Neoantigen selection in NSCLC presents additional challenges because both the quantity and biological quality of potential targets vary substantially across molecular subtypes. Although smoking-associated NSCLC may contain numerous candidate mutations, the therapeutically relevant vaccine target space can be considerably narrower in low-TMB or oncogene-driven tumors. Moreover, the absolute number of predicted neoantigens is unlikely to be sufficient as a selection criterion. Clonality, mutant-allele expression, antigen processing, HLA presentation, and spatial conservation across tumor regions are likely to be more informative determinants of therapeutic value. McCann et al. demonstrated the technical feasibility of identifying personalized vaccine candidates in TMB-low NSCLC and constructing a vaccine targeting 18 tumor mutations. Importantly, this proof-of-concept study used a modified vaccinia Ankara vector rather than an mRNA platform and therefore supports the feasibility of personalized neoantigen targeting in low-TMB disease rather than the clinical efficacy of mRNA vaccination itself [21].

The distinction between clonal and subclonal neoantigens is particularly relevant in NSCLC. Clonal neoantigens, which are present across a larger proportion of malignant cells, are more likely to provide stable and broadly distributed immune targets than subclonal neoantigens restricted to only part of the tumor population. In NSCLC and other solid tumors, clonal neoantigen burden has been associated with greater T-cell immunoreactivity and improved sensitivity to immune checkpoint blockade, supporting the prioritization of truncal rather than highly heterogeneous subclonal targets in personalized vaccine design [37]. This distinction is especially important in a tumor type characterized by extensive spatial and temporal heterogeneity, because a highly immunogenic target may still have limited therapeutic value if it is absent from a substantial fraction of malignant clones.

Tumor evolution creates an additional risk that initially attractive vaccine targets may be lost under therapeutic selection pressure. Al Bakir et al. described a patient with EGFR-mutant lung cancer treated with sequential EGFR tyrosine kinase inhibitors and a personalized neopeptide vaccine targeting ten somatic mutations, including an EGFR exon 19 deletion. Despite induction of systemic T-cell reactivity against the targeted driver neoantigen, disease progression was accompanied by expansion of a pre-existing EGFR-wild-type clone and loss of the vaccine-targeted EGFR alteration [38]. These findings illustrate how therapeutic and immune selection can reshape tumor clonal architecture and permit the outgrowth of malignant populations that no longer express a previously dominant vaccine target. Because this observation was derived from a single patient and INTerpath-009 specifically excludes tumors in which EGFR-directed therapy is indicated, it should be interpreted as evidence of a broader evolutionary principle rather than as a direct predictor of intismeran autogene performance in the phase III population.

**Figure 3:**
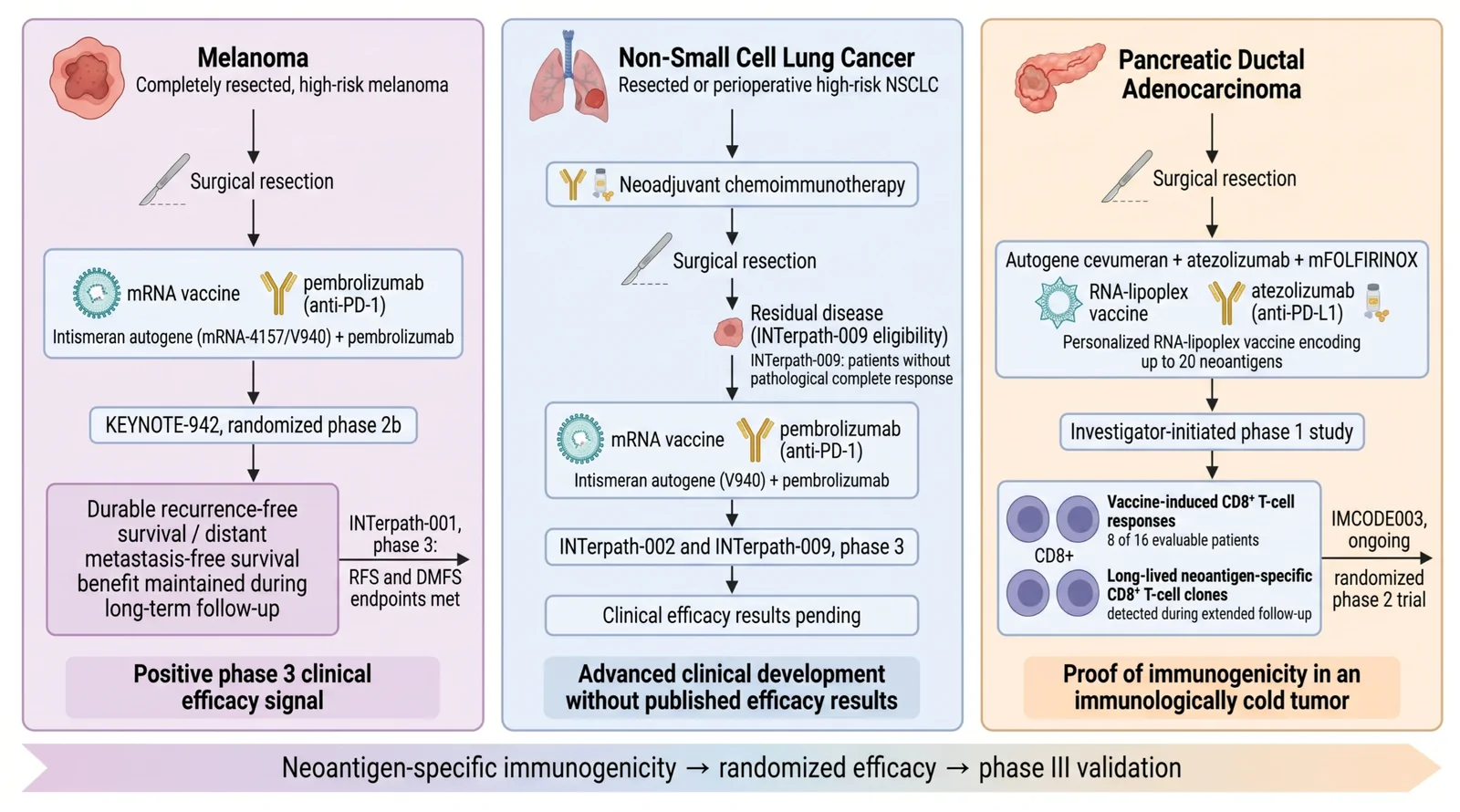
Comparative clinical maturity of personalized mRNA neoantigen vaccine strategies across melanoma, non-small cell lung cancer, and pancreatic ductal adenocarcinoma. Melanoma currently provides the strongest randomized clinical evidence for intismeran autogene combined with pembrolizumab, including the positive phase III INTerpath-001 readout demonstrating significant improvements in RFS and DMFS. In NSCLC, INTerpath-002 and INTerpath-009 are evaluating intismeran autogene plus pembrolizumab in complementary perioperative phase III settings, with efficacy results still pending. In resected PDAC, autogene cevumeran has demonstrated durable vaccine-induced neoantigen-specific T-cell responses within a postoperative multimodal regimen, while randomized phase II evaluation in IMCODE003 remains ongoing. Together, these disease settings illustrate a translational continuum from vaccine immunogenicity to randomized efficacy and phase III validation [8,9,10,11,18,32,33,39] (Created in BioRender.com; Science Suite Inc. dba BioRender, Toronto, ON, Canada).

Taken together, the NSCLC setting highlights a central challenge for personalized cancer vaccination: immunogenicity must be matched by target persistence. Successful vaccine design is therefore likely to require prioritization of expressed, truncal, spatially conserved, and evolutionarily stable neoantigens rather than reliance on predicted HLA-binding affinity alone. Longitudinal circulating tumor DNA analysis, multiregional sequencing, and assessment of clonal neoantigen burden may help determine whether vaccine-targeted alterations remain represented in residual and recurrent disease [15,37,38]. Correlative analyses incorporating smoking history, TMB, oncogenic driver status, HLA genotype, antigen-presentation competence, pathological response, and postoperative molecular residual disease will therefore be critical for interpreting the INTerpath program and for identifying the biological subgroups most likely to benefit from individualized mRNA vaccination [15,34]. In this respect, NSCLC may provide a particularly informative test of whether precision neoantigen selection can overcome tumor heterogeneity sufficiently to convert vaccine-induced immune responses into durable clinical benefit. The relative clinical maturity of the melanoma, NSCLC, and pancreatic ductal adenocarcinoma programs is summarized in Figure 3.

### 3.3. Pancreatic Ductal Adenocarcinoma: Inducing Durable Immunity in an Immunologically Cold Tumor

Pancreatic ductal adenocarcinoma (PDAC) represents a particularly demanding setting for personalized mRNA neoantigen vaccination because it is generally characterized by a relatively low somatic mutation burden, limited baseline T-cell infiltration, and a profoundly immunosuppressive tumor microenvironment [11,18]. Nevertheless, spontaneous neoantigen-specific T-cell responses have been observed in a subset of long-term survivors, indicating that PDAC is not intrinsically refractory to immune recognition [40]. This observation provided a biological rationale for evaluating autogene cevumeran, an individualized uridine mRNA-lipoplex vaccine encoding patient-specific neoantigens, in patients with surgically resected PDAC. In the investigator-initiated phase I study, the postoperative regimen consisted of atezolizumab followed by vaccine priming, adjuvant mFOLFIRINOX, and a subsequent vaccine booster. The strategy was designed to induce de novo neoantigen-specific T-cell responses in the minimal residual disease setting while PD-L1 blockade supported T-cell activity and chemotherapy addressed occult micrometastatic disease [18].

An additional barrier specific to PDAC is its prominent desmoplastic stroma. Dense extracellular matrix deposition together with heterogeneous cancer-associated fibroblast populations can influence vascular perfusion, immune-cell trafficking, and the spatial accessibility of malignant cells to effector lymphocytes. This stromal architecture is therefore relevant not only to conventional drug resistance but also to vaccination: even when a personalized vaccine successfully expands circulating neoantigen-specific T cells, their therapeutic activity may remain limited if stromal organization restricts T-cell entry into tumor nests or sustains local immunosuppressive signaling. In particular, fibroblast-associated chemokine programs and extracellular-matrix remodeling have been implicated in CD8^+^ T-cell exclusion in PDAC. Accordingly, the durable peripheral T-cell responses observed after autogene cevumeran should not be interpreted independently of the stromal context in which these cells must ultimately recognize residual malignant cells. Strategies that normalize, rather than indiscriminately ablate, the PDAC stroma may therefore represent a biologically plausible means of improving the translation of vaccine immunogenicity into tumor control; however, this concept remains to be established prospectively in personalized mRNA vaccine trials [41].

Among the 16 vaccinated patients evaluable for immunogenicity, eight developed high-magnitude vaccine-induced T-cell responses against at least one encoded neoantigen. These responses were not detectable before vaccination, and vaccine-expanded lymphocytes reached substantial frequencies in peripheral blood, including polyfunctional neoantigen-specific CD8^+^ effector T cells. At the initial median follow-up of approximately 18 months, median recurrence-free survival (RFS) was not reached among vaccine responders, whereas non-responders had a median RFS of 13.4 months [18]. Importantly, responders and non-responders did not differ simply in the number of nonsynonymous mutations or vaccine-encoded neoantigens. This observation reinforces the concept that neoantigen quality, presentation, and immune recognition may be more informative than mutation quantity alone when determining the biological effectiveness of individualized vaccination. Even tumors with relatively low mutational burdens may therefore contain a restricted but therapeutically meaningful neoantigen repertoire [18]. However, because the trial was small, non-randomized, and not designed to establish treatment efficacy, the association between vaccine-induced immunity and delayed recurrence should be interpreted as hypothesis-generating rather than causal.

Extended follow-up has provided particularly important evidence regarding the durability and functional persistence of vaccine-induced immunity. At a median follow-up of 3.2 years, median RFS remained unreached among the eight immune responders compared with 13.4 months among the eight non-responders (*p* = 0.007; HR, 0.14; 95% CI, 0.03–0.59). Approximately 86% of vaccine-induced CD8^+^ T-cell clones per patient remained detectable at substantial frequencies approximately three years after vaccination. These clones had an average estimated lifespan of 7.7 years and retained neoantigen-specific effector activity while acquiring cytotoxic and tissue-resident memory-like phenotypic characteristics [11]. These findings are especially relevant in PDAC because they demonstrate that an immunologically cold tumor does not necessarily preclude the generation of durable, functionally persistent vaccine-induced cellular immunity.

The longitudinal analysis also provided evidence consistent with vaccine-associated immunoediting. The two immune responders who subsequently experienced recurrence exhibited fewer persisting vaccine-induced T cells, while recurrent tumors showed depletion of cancer-cell clones carrying vaccine-targeted neoantigens [11]. These observations suggest that personalized vaccination can exert measurable immune selection pressure on PDAC and further emphasize the importance of targeting clonally conserved and evolutionarily stable neoantigens. At the same time, the study does not establish that vaccine-induced immunity itself caused the observed differences in recurrence because treatment was multimodal and the immune-responder comparison was not randomized. The principal contribution of the phase I program is therefore not proof of clinical efficacy, but demonstration that individualized mRNA vaccination can generate de novo CD8^+^ T-cell populations with substantial magnitude, multiyear persistence, and durable effector function in a tumor type traditionally considered poorly immunogenic [11].

The PDAC experience also illustrates an important distinction between vaccine immunogenicity and therapeutic efficacy. Durable expansion of vaccine-specific T-cell clones demonstrates successful immune priming, but clinical benefit ultimately depends on whether these populations can continue to recognize clonally relevant tumor targets, traffic effectively to sites of residual disease, and resist ongoing immune suppression and tumor evolution. In this respect, PDAC provides a particularly informative model for examining whether the quality and persistence of vaccine-induced immunity can compensate for an unfavorable baseline immune microenvironment.

Clinical efficacy is now being evaluated prospectively in IMCODE003 (NCT05968326), an ongoing randomized, open-label phase II trial comparing adjuvant autogene cevumeran plus atezolizumab and mFOLFIRINOX with mFOLFIRINOX alone in patients with completely resected PDAC and no evidence of disease after surgery [39]. The study has an estimated enrollment of 260 participants, with disease-free survival as the primary endpoint. As of August 2026, the trial remains recruiting and no efficacy results have been reported [39]. Confirmation in this controlled setting will be essential to determine whether the durable vaccine-induced immunity observed in the phase I cohort translates into a clinically meaningful reduction in postoperative recurrence.

### 3.4. Platform Comparison

Although intismeran autogene combined with pembrolizumab currently anchors the most mature clinical evidence for personalized mRNA neoantigen vaccination in melanoma and represents one of the most advanced development programs in NSCLC, the broader field encompasses several individualized vaccine architectures that differ in nucleic-acid format, delivery strategy, immune-modulating partner, and stage of clinical development [5,17,42]. These platforms share the central objective of generating patient-specific T-cell responses against tumor-derived neoantigens, but they differ substantially in how neoantigen payloads are encoded, delivered, and integrated with other immune-modulating therapies.

Comparison across platforms therefore requires consideration not only of vaccine format, but also of disease setting, tumor burden, treatment timing, and the biological context in which vaccine-induced immunity is generated. Cross-trial comparisons should therefore be interpreted cautiously. The most mature positive efficacy data derive from resected melanoma, where vaccination is administered in a minimal-residual-disease setting with relatively low macroscopic tumor burden and established sensitivity to PD-1 blockade. By contrast, NSCLC encompasses substantial molecular and immune heterogeneity, and the major randomized vaccine trials remain ongoing in perioperative populations defined by different treatment sequences and residual-disease states [9,10]. PDAC presents a distinct challenge characterized by low baseline immunogenicity, stromal and myeloid immune suppression, and multimodal postoperative treatment, making it particularly difficult to isolate the contribution of vaccination from atezolizumab and mFOLFIRINOX in non-randomized studies [11,18,39]. Accordingly, differences in observed outcomes across these malignancies should not be attributed to vaccine platform alone but interpreted in the context of disease biology, tumor burden, timing of vaccination, and concomitant therapy. Because the clinically advanced studies combine vaccination with checkpoint blockade, the observed treatment effect reflects the combination strategy; although randomized comparator arms can estimate the incremental benefit associated with adding vaccination, they do not establish that vaccine-induced immune remodeling is the sole mechanism responsible for the clinical outcome.

One alternative strategy uses a heterologous prime-boost architecture combining a chimpanzee adenoviral vector with self-amplifying mRNA encoding the same individualized neoantigen set. In a phase I trial involving patients with advanced metastatic solid tumors, this approach was feasible and generally well tolerated and generated vaccine-induced T-cell responses, providing proof of concept that personalized neoantigen vaccination can be implemented through sequential viral-vector and self-amplifying RNA delivery rather than conventional mRNA alone [42]. The study primarily established safety, immunogenicity, and feasibility and should therefore be interpreted as an early-phase demonstration rather than evidence of clinical efficacy.

Autogene cevumeran represents another clinically advanced individualized RNA platform. Its development illustrates a distinct RNA-lipoplex approach and has generated particularly informative evidence in resected PDAC, where vaccine-induced neoantigen-specific T-cell populations persisted for several years following treatment [11,18]. The ongoing randomized phase II IMCODE003 trial is now testing whether this immunogenicity can translate into improved disease-free survival when autogene cevumeran is combined with atezolizumab and mFOLFIRINOX after PDAC resection [39].

Autogene cevumeran was also evaluated with pembrolizumab in the randomized phase II IMCODE001 study (NCT03815058) in patients with previously untreated advanced melanoma. The study was completed without demonstrating the anticipated improvement in its primary efficacy endpoint of progression-free survival [43]. This finding is particularly instructive because biological immunogenicity and clinical efficacy diverged within the same development program. The ability of autogene cevumeran to induce neoantigen-specific T-cell responses in earlier studies did not translate into a demonstrable PFS advantage in this randomized advanced-melanoma setting. Differences in disease stage, macroscopic tumor burden, baseline immune suppression, antigenic heterogeneity, and treatment context may all have contributed. The result therefore cautions against using vaccine-induced immune responses as a surrogate for clinical benefit and emphasizes the need for randomized disease-specific efficacy endpoints.

Beyond the PDAC and melanoma programs, autogene cevumeran has undergone broad phase I evaluation in patients with advanced solid tumors. In NCT03289962, the vaccine was evaluated as monotherapy in 30 patients and in combination with atezolizumab in 183 patients with previously treated advanced solid tumors. Neoantigen-specific CD4^+^ and/or CD8^+^ T-cell responses were detected in a substantial proportion of treated patients and remained detectable longitudinally, demonstrating broad immunogenicity across multiple tumor types [17]. However, the study was primarily designed to establish safety and immunogenicity, and the observed clinical activity should therefore be regarded as preliminary.

The field also includes individualized neoantigen vaccines based on non-mRNA technologies. In resected clear-cell renal cell carcinoma, a personalized peptide-based neoantigen vaccine induced antitumor immune responses in all nine treated patients in a phase I study despite the relatively low mutational burden characteristic of this malignancy [44]. Although this finding does not provide direct evidence for an mRNA platform, it reinforces a broader principle relevant to personalized vaccine design: neoantigen quality and biological relevance may be more important than mutation quantity alone.

Taken together, these programs demonstrate that personalized neoantigen vaccination is a platform concept rather than a single therapeutic technology. Current strategies range from conventional synthetic mRNA and RNA-lipoplex formulations to self-amplifying RNA, viral-vector prime-boost regimens, and individualized peptide vaccines [5,17,42,44]. However, their levels of clinical maturity and the biological questions they address differ substantially. Intismeran autogene currently provides the strongest randomized efficacy signal in combination with checkpoint blockade, whereas autogene cevumeran has demonstrated broad immunogenicity across advanced solid tumors and durable vaccine-induced cellular immunity in resected PDAC [11,17,18]. At the same time, the negative randomized IMCODE001 result in advanced melanoma demonstrates that vaccine immunogenicity alone is insufficient to guarantee clinical benefit [43], while randomized evaluation of autogene cevumeran in resected PDAC remains ongoing [39].

The emerging pattern across platforms therefore suggests that successful personalized vaccination depends on more than vaccine architecture alone. Disease stage, residual tumor burden, target clonality, antigen-presentation competence, immune-contexture characteristics, and the durability of vaccine-induced T-cell responses are likely to shape whether molecularly precise vaccine design can be translated into meaningful clinical benefit. Other individualized platforms remain primarily at the feasibility and immunogenicity stage. The principal characteristics, supporting evidence, and current clinical status of selected platforms are summarized in Table 1.

These studies also underscore the need to distinguish vaccine immunogenicity from clinical efficacy. Immunogenicity endpoints, including the induction, magnitude, breadth, persistence, or clonality of neoantigen-specific T-cell responses, demonstrate biological activity but do not by themselves establish therapeutic benefit. Clinically meaningful efficacy requires improvement in disease-centered outcomes such as recurrence-free survival (RFS), disease-free survival (DFS), progression-free survival (PFS), distant metastasis-free survival (DMFS), overall survival (OS), or pathological response. This distinction is illustrated by the contrast between KEYNOTE-942, in which intismeran autogene plus pembrolizumab improved RFS and DMFS in resected high-risk melanoma, and IMCODE001, in which the addition of autogene cevumeran did not demonstrate the anticipated PFS benefit in advanced melanoma [7,8,43]. In resected PDAC, durable vaccine-induced T-cell responses are biologically compelling, but randomized evidence that these responses improve DFS remains pending from IMCODE003 [11,18,39].

## 4. Challenges and Limitations

The clinical translation of personalized mRNA neoantigen vaccination depends on overcoming a series of interrelated technological, biological, and implementation barriers. These challenges extend beyond vaccine manufacturing alone and include the accuracy of neoantigen selection, temporal and spatial tumor heterogeneity, antigen-presentation competence, immune suppression within the tumor microenvironment, and the absence of validated biomarkers capable of identifying patients most likely to benefit. Importantly, these limitations are not independent. Delays in manufacturing can alter the temporal relevance of selected targets, tumor evolution can render initially prioritized neoantigens obsolete, and robust systemic T-cell responses may fail to produce clinical benefit when antigen presentation or intratumoral immune access is impaired.

### 4.1. Manufacturing Timelines and Logistics

A defining challenge of personalized mRNA neoantigen vaccination is that each therapeutic product must be generated de novo from an individual patient’s tumor. The workflow requires coordinated tissue acquisition, tumor and matched-normal sequencing, transcriptomic analysis, bioinformatic neoantigen prediction and prioritization, construct design, good manufacturing practice (GMP)-compliant production, quality control, and product release. Even when optimized, this process generally requires several weeks, creating a potential mismatch between manufacturing timelines and the therapeutic needs of patients with rapidly progressive disease [20]. Practical attrition can occur at multiple points in this workflow. Limited or low-quality tumor tissue may yield insufficient material for reliable sequencing or neoantigen calling, while low tumor purity, degraded nucleic acids, or inadequate RNA expression can reduce confidence in candidate selection. Even after successful target identification, individualized manufacture may fail to meet release specifications or may be delayed by production and quality-control requirements. These risks are clinically relevant because patients with rapidly progressive disease may deteriorate or lose eligibility before vaccine administration. In addition, each patient-specific product requires individualized documentation, traceability, release testing, and regulatory oversight, creating operational demands that extend well beyond those of conventional batch-manufactured therapeutics [20].

Centralized manufacturing models introduce additional operational complexity by requiring efficient transfer of molecular data, coordination among sequencing, computational, manufacturing, and clinical facilities, and reliable distribution of the final product to the treating center [5,20]. For mRNA-based platforms, formulation stability and cold-chain requirements may add further logistical constraints. These issues are particularly relevant for individualized therapies because manufacturing delays affect not only treatment timing but also the biological relevance of the selected vaccine targets if substantial tumor evolution occurs between tissue acquisition and vaccine administration.

The challenge becomes especially pronounced in perioperative settings, where vaccination must be integrated with surgery, chemotherapy, and immune checkpoint blockade within predefined therapeutic windows. Delays in tissue processing, neoantigen prioritization, manufacturing, or product release could therefore postpone therapy or compromise eligibility. Shortening the interval from tissue acquisition to vaccine administration remains a major translational objective and will likely require greater automation of sequencing and bioinformatic pipelines, streamlined manufacturing workflows, and potentially decentralized or modular production strategies capable of maintaining regulatory-grade quality control [20]. Thus, manufacturing speed should be regarded not only as a logistical variable but also as a biological determinant of whether the vaccine continues to target the clinically relevant tumor architecture at the time of treatment.

### 4.2. Cost and Accessibility

The individualized nature of personalized mRNA vaccines also creates substantial economic barriers. Unlike off-the-shelf anticancer agents, each vaccine requires patient-specific sequencing, computational analysis, construct generation, manufacturing, quality control, and product-release testing. This limits the economies of scale that characterize conventional batch-produced therapeutics and is likely to contribute to high per-patient costs, particularly when individualized vaccination is combined with checkpoint inhibitors, chemotherapy, or other components of multimodal treatment regimens [20].

The accessibility challenge extends beyond the direct cost of vaccine manufacture. Successful implementation requires infrastructure for high-quality tumor sequencing, HLA typing, bioinformatic analysis, specialized manufacturing, quality assurance, and multidisciplinary clinical coordination. Such resources remain concentrated in well-resourced academic and biotechnology environments and may be difficult to reproduce across lower-resource healthcare systems [20].

Consequently, broader clinical adoption will depend not only on demonstration of therapeutic efficacy but also on the development of scalable production models, sustainable reimbursement frameworks, and infrastructure capable of supporting individualized genomic medicine across diverse healthcare settings. Without such advances, personalized neoantigen vaccination risks becoming technically feasible but unevenly accessible, particularly when compared with standardized immunotherapeutic approaches.

### 4.3. Tumor Heterogeneity and Immune Escape

Intratumoral heterogeneity represents a fundamental biological limitation to personalized neoantigen vaccination. Neoantigens identified from a single biopsy or resected specimen may not be uniformly expressed across all malignant subclones, and vaccine-induced immune pressure may preferentially eliminate antigen-positive populations while permitting antigen-negative or weakly recognized subclones to persist and expand. Experimental evidence demonstrates that the spatial and clonal architecture of neoantigen expression can strongly influence immune recognition and can facilitate immune escape when antigen expression is heterogeneous [24].

Loss or impairment of antigen presentation constitutes a second major escape mechanism. Even when vaccination successfully primes high-affinity tumor-reactive T cells, malignant cells may evade recognition through reduced HLA expression, defects in components of the antigen-processing machinery, or other alterations that interfere with peptide presentation [23]. Because cognate peptide-HLA presentation is required for effective recognition by vaccine-induced T cells, defects in antigen presentation can render an otherwise immunogenic and clonally conserved neoantigen therapeutically irrelevant.

Tumor evolution further complicates target selection because neoantigen architecture is not necessarily stable over the course of treatment. Therapeutic and immune selection pressures can reshape clonal composition and lead to loss of previously dominant vaccine targets. In EGFR-mutant lung cancer, Al Bakir et al. demonstrated loss of a targeted clonal driver neoantigen under combined EGFR tyrosine kinase inhibitor and immune selection pressure, illustrating how treatment can favor expansion of malignant populations that no longer carry the targeted alteration [38]. These observations reinforce the importance of prioritizing expressed, truncal, and evolutionarily stable neoantigens and suggest that longitudinal molecular monitoring may be necessary to determine whether vaccine targets remain represented in residual or recurrent disease [24,37,38].

Accordingly, neoantigen selection should be viewed as a dynamic rather than purely static process. A target identified at diagnosis may not retain the same therapeutic relevance after surgery, systemic therapy, or immune-mediated selection. Multiregional sampling, longitudinal circulating tumor DNA analysis, and reassessment of clonal architecture may therefore become increasingly important for linking precision vaccine design to the evolving molecular landscape of an individual tumor.

### 4.4. Immunosuppressive Tumor Microenvironment

Even when neoantigen selection, vaccine manufacturing, T-cell priming, and antigen presentation are successful, the tumor microenvironment may remain sufficiently immunosuppressive to limit clinical efficacy. High densities of regulatory T cells, immunosuppressive myeloid populations, inhibitory cytokine networks, metabolic constraints, and stromal barriers can impair T-cell trafficking and effector function despite concurrent checkpoint blockade. This limitation is particularly relevant in poorly infiltrated or immunologically “cold” tumors [23,25,35].

NSCLC provides a clinically important example because immune resistance can be driven by both tumor-intrinsic and microenvironmental factors, including exclusion or dysfunction of effector lymphocytes and enrichment of suppressive stromal and myeloid compartments. Strategies aimed at converting poorly infiltrated tumors into more inflamed and T-cell-accessible states are therefore being investigated as a means of improving responsiveness to checkpoint inhibition [35].

For personalized mRNA vaccination, this distinction is critical because expansion of circulating neoantigen-specific T cells does not necessarily imply effective intratumoral immunity. Vaccine-induced lymphocytes must reach the tumor, recognize the relevant peptide-HLA complex, retain effector competence, and operate within a metabolically and immunologically restrictive environment. In selected tumor types, additional interventions targeting myeloid suppression, stromal exclusion, metabolic inhibition, or non-redundant checkpoint pathways may therefore be required to complement vaccination and PD-1/PD-L1 blockade [35,45].

The therapeutic objective should consequently extend beyond generating a large systemic vaccine response toward creating an immune context in which vaccine-expanded cells can remain functionally active at the site of residual or established disease. This distinction may help explain why strong vaccine immunogenicity does not uniformly translate into clinical benefit.

### 4.5. Biomarker Needs and Patient Selection

Reliable biomarkers for identifying patients most likely to benefit from personalized mRNA neoantigen vaccination combined with checkpoint blockade remain poorly defined [13,15]. Although PD-L1 expression and tumor mutational burden have established or emerging roles in guiding checkpoint inhibitor therapy in selected clinical settings, neither directly measures the probability that a personalized vaccine will generate functionally relevant antitumor immunity [13,15].

TMB may increase the number of mutations available for neoantigen discovery, but mutation burden alone does not determine whether these alterations are expressed, processed, presented by the patient’s HLA molecules, clonally conserved, or recognized by functional T-cell repertoires [15]. Similarly, a computationally predicted high-affinity peptide may have limited therapeutic relevance if the corresponding mutation is subclonal, poorly expressed, inefficiently processed, or lost during tumor evolution.

More informative biomarker strategies will therefore likely require integration of multiple biological layers, including neoantigen clonality, mutant-allele expression, predicted and experimentally validated HLA presentation, antigen-presentation competence, baseline immune infiltration, and on-treatment expansion and persistence of vaccine-specific T-cell populations. Computational tools such as pVACview already facilitate multidimensional neoantigen prioritization by integrating variant-, transcript-, and peptide-level information, but no single computational score has yet been validated as a clinically reliable predictor of benefit from personalized vaccination [13].

Longitudinal immune monitoring may be equally important. Measurements of vaccine-specific T-cell expansion, persistence, phenotypic state, and T-cell receptor repertoire remodeling could help distinguish transient immunogenicity from sustained biologically meaningful immune responses [11,29]. The development of standardized immune-monitoring assays and composite biomarkers integrating genomic, transcriptomic, immunopeptidomic, tumor-microenvironment, and longitudinal immune-response data will therefore be critical both for patient selection and for interpreting future randomized trials [11,13,29].

Ultimately, the most useful biomarker is unlikely to be a single molecular variable. Personalized vaccination itself is a multistep biological process, and predictive models may need to capture the complete sequence from target existence and presentation to T-cell generation, intratumoral access, functional persistence, and continued target expression. Such multidimensional biomarker strategies may be essential for distinguishing patients in whom vaccine immunogenicity is likely to translate into durable therapeutic benefit from those in whom one or more downstream biological bottlenecks remain dominant.

Importantly, biomarkers used clinically for checkpoint-inhibitor selection should not be assumed to have validated predictive value for personalized neoantigen vaccination. At present, no biomarker has been prospectively established as a clinically validated predictor of benefit from personalized mRNA neoantigen vaccination. Parameters such as neoantigen clonality, mutant-allele expression, experimentally validated HLA presentation, vaccine-specific T-cell expansion, T-cell receptor clonality, and immune-contexture features should therefore be regarded as exploratory or hypothesis-generating until validated prospectively in randomized studies [15,20].

## 5. Future Directions

Much of the clinical development of personalized mRNA neoantigen vaccines has focused on the adjuvant setting after surgical resection, where tumor burden is minimal and vaccine-induced immune responses can potentially target residual micrometastatic disease [20,34]. An important future question is whether selected vaccine-checkpoint blockade combinations could also be integrated earlier in the treatment course. The neoadjuvant setting is immunologically attractive because the intact primary tumor provides a broader antigenic source during immune priming, although the individualized manufacturing interval remains an important practical constraint [34]. This issue is particularly relevant in NSCLC, where neoadjuvant chemoimmunotherapy has become an established component of perioperative treatment and patients with residual viable disease after such therapy are now being studied in postoperative vaccine-based consolidation strategies, including INTerpath-009 [9,34]. Future studies should therefore define the optimal temporal relationship among tumor sampling, neoantigen identification, vaccine manufacture, surgery, chemotherapy, and checkpoint blockade rather than assuming that one treatment sequence will be universally superior.

The timing question is closely linked to tumor burden and evolutionary dynamics. Minimal residual disease may provide a favorable setting in which vaccine-expanded T cells encounter fewer malignant cells and reduced tumor-mediated immunosuppression, whereas advanced disease may present greater antigenic diversity but also more extensive heterogeneity, immune dysfunction, and opportunities for clonal escape. Clinical development should therefore consider disease stage and tumor burden as biological variables that can influence the translation of vaccine immunogenicity into therapeutic efficacy, rather than treating them solely as conventional trial-stratification factors.

Beyond PD-1/PD-L1 blockade, personalized mRNA vaccination could potentially be combined with agents targeting additional, non-redundant mechanisms of immune suppression. Combination strategies involving distinct checkpoint pathways or other regulators of T-cell activity have been proposed as a means of overcoming primary and acquired resistance to PD-1/PD-L1 inhibition [45]. One emerging example is the adenosine A2A receptor pathway, which can suppress T-cell function in metabolically stressed and hypoxic tumor microenvironments [46]. A2A receptor antagonism has therefore been proposed as a complementary strategy for sustaining neoantigen-specific T-cell activity induced by mRNA vaccination, either alongside or independently of PD-1/PD-L1 blockade [46]. Additional interventions targeting immunosuppressive myeloid populations, stromal exclusion, metabolic constraints, or alternative checkpoint pathways may be particularly relevant in tumors such as NSCLC and PDAC, in which effective T-cell priming alone may be insufficient to overcome a strongly suppressive tumor microenvironment [35]. These approaches remain investigational and will require prospective evidence demonstrating that additional immune modulation improves clinical outcomes without producing disproportionate toxicity.

A second major area of development will be the refinement of neoantigen discovery and prioritization. Current computational pipelines rely on multiple imperfect predictors, including mutation detection, transcript expression, HLA binding, peptide processing, and immunogenicity estimates. Proteogenomic approaches incorporating mass spectrometry-based immunopeptidomics can provide direct evidence that predicted neoantigens are actually presented by tumor cells or relevant antigen-presenting cells and may therefore increase the precision of vaccine-target selection [12]. In parallel, artificial intelligence-assisted models are being developed to integrate sequence, structural, expression, and immunological features into more sophisticated estimates of neoantigen quality [14]. Interactive platforms such as pVACview may further facilitate systematic candidate review by integrating variant-, transcript-, and peptide-level evidence into a clinically interpretable prioritization workflow [13].

The objective of these advances should not simply be to increase the number of predicted neoantigens incorporated into each vaccine, but to improve identification of targets that are expressed, clonally conserved, efficiently processed and presented, and genuinely immunogenic [3,12,13,15]. Future vaccine design may therefore increasingly shift from maximizing antigen quantity toward optimizing antigen quality and evolutionary stability. Longitudinal reassessment of vaccine targets may also become important in settings where systemic therapy or immune selection alters tumor clonal architecture over time.

Advances in target selection may facilitate expansion into malignancies traditionally considered less favorable for personalized vaccination because of relatively low mutational burden or limited baseline immunogenicity. Early clinical studies already demonstrate that meaningful neoantigen-specific immunity can be generated in such settings. In resected clear-cell renal cell carcinoma, individualized neoantigen vaccination induced measurable antitumor immune responses despite the comparatively low mutational burden of the disease [44]. Similarly, autogene cevumeran generated de novo CD8^+^ T-cell populations with multiyear persistence and preserved effector function in resected PDAC [11]. These observations suggest that neoantigen quality, clonality, expression, presentation, and immune recognition may ultimately be more important than mutation quantity alone when determining whether a tumor is suitable for individualized vaccination [11,15,44].

Future clinical trials should also incorporate standardized translational endpoints alongside conventional measures such as recurrence-free, disease-free, and overall survival. Longitudinal quantification of vaccine-specific T-cell expansion, persistence, functional phenotype, T-cell receptor clonality, and epitope spreading could help establish whether a vaccine has generated the intended biological response and may identify early correlates of subsequent clinical benefit [8,11,29]. Circulating tumor DNA and other measures of molecular residual disease may provide an additional means of relating vaccine-induced immunity to changes in residual tumor burden and tumor evolution [15,34].

Because tumor mutational burden alone does not reliably capture neoantigen quality or predict vaccine immunogenicity, composite biomarker strategies integrating genomic, transcriptomic, immunopeptidomic, tumor-microenvironment, and longitudinal immune-monitoring data may ultimately prove more informative for patient selection and trial interpretation [15]. Prospective validation of such biomarkers will be essential before they can be used to guide treatment decisions or adaptive trial designs.

Taken together, the next phase of personalized mRNA neoantigen vaccine development should move beyond the question of whether a vaccine can induce an immune response toward determining when, where, and in whom that response is most likely to remain therapeutically effective. Progress will therefore depend on coordinating precision target selection, optimized treatment timing, control of tumor-mediated immune suppression, longitudinal monitoring of tumor evolution, and biomarker-guided patient selection within a unified translational framework.

## 6. Conclusions

Personalized mRNA neoantigen vaccination and immune checkpoint blockade address complementary limitations of antitumor immunity. Personalized vaccination can expand the magnitude and breadth of tumor-reactive T-cell responses, whereas checkpoint blockade can preserve or restore the functional competence of these lymphocytes within an immunosuppressive tumor microenvironment [3,4]. The strongest clinical signal has emerged in resected high-risk melanoma, where intismeran autogene combined with pembrolizumab demonstrated durable improvements in recurrence-free and distant metastasis-free survival compared with pembrolizumab alone, with treatment effects remaining evident after approximately five years of follow-up [7,8]. Phase III development in NSCLC will determine whether this benefit can be reproduced in a more biologically heterogeneous malignancy [9,10], while studies in PDAC demonstrate that durable neoantigen-specific cellular immunity can be generated even in tumors traditionally considered poorly immunogenic [11,18].

However, the available evidence also demonstrates that vaccine immunogenicity and clinical efficacy are not equivalent. The therapeutic success of personalized vaccination depends on a sequence of interdependent biological events: selection of relevant and clonally conserved neoantigens, efficient antigen expression and presentation, generation of functional tumor-reactive T cells, successful trafficking into the tumor microenvironment, preservation of effector activity, and continued expression of the targeted antigenic landscape. Failure at any of these stages can limit clinical benefit despite successful vaccine-induced immune priming.

The eventual clinical role of personalized mRNA neoantigen vaccination will therefore depend on overcoming substantial technological and biological barriers, including manufacturing time and cost, intratumoral heterogeneity, antigen-presentation defects, immune escape, and the absence of validated predictive biomarkers [20,23,24]. Improvements in neoantigen prioritization, scalable manufacturing, longitudinal monitoring of tumor evolution, and integration of genomic, immunopeptidomic, and immune-response biomarkers will be essential for translating vaccine-induced immunity into durable clinical benefit [12,13,20].

Rather than viewing personalized mRNA vaccination as a stand-alone platform, its future development is likely to depend on matching molecularly precise antigen selection with the immune and evolutionary state of each tumor. If ongoing randomized trials confirm the efficacy signals observed in early- and mid-phase studies, personalized mRNA neoantigen vaccination combined with appropriately selected immune-modulating strategies could become an important component of biomarker-guided multimodal immunotherapy for selected patients with solid tumors.

## Figures and Tables

**Table 1:** Comparative clinical evidence for selected personalized neoantigen vaccine platforms and trials.

Platform/Trial	Tumor Type/Clinical Setting	Phase/Sample Size	Treatment Regimen	Primary or Major Endpoint(s)	Key Immunogenicity/Efficacy Finding	Principal Limitation/Interpretation	References
Intismeran autogene (mRNA-4157/V940), KEYNOTE-942 (NCT03897881)	Completely resected high-risk stage III–IV melanoma; adjuvant setting	Randomized phase IIb; *N* = 157 (107 combination; 50 pembrolizumab alone)	Intismeran autogene + pembrolizumab vs. pembrolizumab alone	RFS; DMFS; exploratory OS	Combination produced durable improvement in RFS and DMFS. At approximately 5 years, RFS HR = 0.510 (95% CI, 0.294–0.887) and DMFS HR = 0.411 (95% CI, 0.200–0.843). OS showed a favorable but immature trend (HR = 0.471; 95% CI, 0.165–1.345). Increased T-cell receptor clonality and emergence of novel clonotypes were also observed with the combination.	Relatively small, open-label phase IIb study; OS remains immature. Because both arms received pembrolizumab, the randomized comparison estimates the incremental benefit of adding intismeran but does not establish vaccine-induced immune remodeling as the sole mechanism of benefit.	[7,8]
Intismeran autogene, INTerpath-001/V940-001 (NCT05933577)	Completely resected stage IIB–IV cutaneous melanoma; adjuvant setting	Randomized, double-blind phase III; estimated *N* = 1089	Intismeran autogene + pembrolizumab vs. placebo + pembrolizumab	Primary: RFS; key secondary: DMFS; OS among additional secondary endpoints	At the prespecified interim analysis announced in August 2026, the combination met the primary RFS endpoint and key secondary DMFS endpoint, with statistically significant and clinically meaningful improvements over pembrolizumab alone. No new safety signals were reported.	Positive topline phase III result; numerical effect estimates and mature OS data had not yet been publicly reported at the time of this review. Full presentation and peer-reviewed publication remain necessary for detailed interpretation.	[32,33]
Intismeran autogene, INTerpath-002/V940-002 (NCT06077760)	Completely resected stage II, IIIA, or IIIB (N2) NSCLC; adjuvant setting without prior neoadjuvant systemic therapy	Randomized, double-blind phase III; estimated *N* = 868	Intismeran autogene + pembrolizumab vs. placebo + pembrolizumab	Primary: DFS	Phase III efficacy evaluation is ongoing; no efficacy results reported as of August 2026.	Clinical benefit remains unestablished. NSCLC molecular heterogeneity, variable TMB and neoantigen clonality may complicate translation of vaccine immunogenicity into DFS benefit.	[10]
Intismeran autogene, INTerpath-009/V940-009 (NCT06623422)	Resectable stage II–IIIB (N2) NSCLC with residual viable disease/failure to achieve pCR after neoadjuvant pembrolizumab + platinum-based chemotherapy and surgery	Randomized phase III; estimated *N* = 680	Adjuvant pembrolizumab + intismeran autogene vs. pembrolizumab + placebo following neoadjuvant chemoimmunotherapy and surgery	Primary: DFS	No efficacy results reported as of August 2026. Trial specifically tests whether individualized vaccination can improve outcomes in a high-risk residual-disease population.	Complex perioperative treatment sequence makes biological attribution challenging; efficacy of vaccination remains hypothesis-generating until randomized DFS/OS results become available.	[9]
Autogene cevumeran (BNT122/RO7198457), resected PDAC phase I	Surgically resected PDAC; postoperative minimal-residual-disease setting	Phase I; 16 vaccinated patients evaluable for immunogenicity	Surgery → atezolizumab → autogene cevumeran → mFOLFIRINOX → vaccine booster	Vaccine-induced neoantigen-specific T-cell response; feasibility; exploratory RFS	8/16 patients (50%) developed de novo high-magnitude neoantigen-specific T-cell responses. At 18-month follow-up, median RFS was not reached in immune responders versus 13.4 months in non-responders; durable vaccine-expanded T-cell populations were subsequently observed.	Very small, non-randomized cohort. RFS comparison was based on post-treatment immune-response groups and cannot establish vaccine efficacy. Contributions of atezolizumab and mFOLFIRINOX cannot be separated from vaccination.	[11,18]
Autogene cevumeran, IMCODE003 (NCT05968326)	Completely resected PDAC with no evidence of disease after surgery; adjuvant setting	Randomized open-label phase II; estimated *N* = 260	Autogene cevumeran + atezolizumab + mFOLFIRINOX vs. mFOLFIRINOX alone	Primary: DFS; secondary endpoints include OS	Ongoing randomized trial designed to determine whether the strong vaccine-induced immunogenicity observed in phase I translates into improved clinical outcome. No efficacy results reported as of August 2026.	Until randomized DFS results are available, durable vaccine-specific T-cell responses in PDAC should be interpreted as immunogenicity rather than proven clinical efficacy. Combination with atezolizumab also complicates attribution of any future benefit solely to vaccination.	[39]
Autogene cevumeran, IMCODE001 (NCT03815058)	Previously untreated advanced melanoma; macroscopic metastatic/unresectable disease	Randomized open-label phase II; *N* = 131	Autogene cevumeran + pembrolizumab vs. pembrolizumab alone	Primary: PFS	The study did not demonstrate the anticipated improvement in PFS with addition of autogene cevumeran.	Important negative randomized example showing that biological immunogenicity does not necessarily translate into clinical efficacy. Advanced disease burden, tumor heterogeneity, immune suppression and treatment context may limit translation of vaccine-induced T-cell responses into tumor control.	[43]
Autogene cevumeran, NCT03289962	Previously treated locally advanced or metastatic solid tumors across multiple histologies	Phase Ia/Ib; 30 monotherapy + 183 combination patients in the published major cohorts	Autogene cevumeran alone or + atezolizumab	Safety, tolerability and immunogenicity; clinical activity exploratory	Neoantigen-specific CD4^+^ and/or CD8^+^ T-cell responses were induced across multiple tumor types, demonstrating broad biological activity and feasibility.	Early-phase, heterogeneous, non-randomized population; designed primarily for safety and immunogenicity. Clinical efficacy cannot be inferred from immune-response rates.	[17]

Abbreviations: RFS, recurrence-free survival; DMFS, distant metastasis-free survival; HR, hazard ratio; CI, confidence interval; OS, overall survival; NSCLC, non-small cell lung cancer; TMB, tumor mutational burden; PDAC, pancreatic ductal adenocarcinoma.

## Data Availability

No new data were created or analyzed in this study. Data sharing is not applicable to this article.

## Abbreviations

The following abbreviations are used in this manuscript:

A2A	Adenosine A2A receptor
CTLA-4	Cytotoxic T-lymphocyte-associated protein 4
DFS	Disease-free survival
DMFS	Distant metastasis-free survival
GMP	Good Manufacturing Practice
HLA	Human leukocyte antigen
ICB	Immune checkpoint blockade
ICI	Immune checkpoint inhibitor
LNP	Lipid nanoparticle
MHC	Major histocompatibility complex
mRNA	Messenger RNA
NSCLC	Non-small cell lung cancer
OS	Overall survival
pCR	Pathological complete response
PD-1	Programmed cell death protein 1
PD-L1	Programmed death-ligand 1
PDAC	Pancreatic ductal adenocarcinoma
RFS	Recurrence-free survival
TMB	Tumor mutational burden
Treg	Regulatory T cell
WES	Whole-exome sequencing

## References

[1] Zhou X., Ni Y., Liang X., Lin Y., An B., He X., Zhao X. (2022). Mechanisms of Tumor Resistance to Immune Checkpoint Blockade and Combination Strategies to Overcome Resistance. Front. Immunol..

[2] Sun Q., Hong Z., Zhang C., Wang L., Han Z., Ma D. (2023). Immune Checkpoint Therapy for Solid Tumours: Clinical Dilemmas and Future Trends. Signal Transduct. Target. Ther..

[3] Lang F., Schrörs B., Löwer M., Türeci Ö., Sahin U. (2022). Identification of Neoantigens for Individualised Cancer Immunotherapy. Nat. Rev. Drug Discov..

[4] Liu L., Chen J., Zhang H., Ye J., Moore C., Lu C., Fang Y., Fu Y.-X., Li B. (2022). Concurrent Delivery of Immune Checkpoint Blockade Modulates T Cell Dynamics to Enhance Neoantigen Vaccine-Generated Antitumor Immunity. Nat. Cancer.

[5] Floudas C.S., Sarkizova S., Ceccarelli M., Zheng W. (2025). Leveraging mRNA Technology for Antigen Based Immuno-Oncology Therapies. J. Immunother. Cancer.

[6] Lin X., Kang K., Chen P., Zeng Z., Li G., Xiong W., Yi M., Xiang B. (2024). Regulatory Mechanisms of PD-1/PD-L1 in Cancers. Mol. Cancer.

[7] Weber J.S., Carlino M.S., Khattak A., Meniawy T., Ansstas G., Taylor M.H., Kim K.B., McKean M., Long G.V., Sullivan R.J. (2024). Individualised Neoantigen Therapy mRNA-4157 (V940) plus Pembrolizumab versus Pembrolizumab Monotherapy in Resected Melanoma (KEYNOTE-942): A Randomised, Phase 2b Study. Lancet.

[8] Khattak A., Carlino M.S., Meniawy T., Ansstas G., Taylor M.H., Kim K.B., McKean M., Long G.V., Sullivan R.J., Faries M. (2026). Intismeran Autogene Plus Pembrolizumab Versus Pembrolizumab Alone in High-Risk Resected Melanoma: 5-Year Update of the Randomized Phase IIb KEYNOTE-942 Study. J. Clin. Oncol..

[9] Merck Sharp & Dohme LLC (2026). A Phase 3 Randomized Double-Blind Study of Adjuvant Pembrolizumab With or Without V940 in Participants with Resectable Stage II to IIIB (N2) NSCLC Not Achieving pCR After Receiving Neoadjuvant Pembrolizumab with Platinum-Based Doublet Chemotherapy (INTerpath-009). https://www.istitutotumori.mi.it/en/clinical-trials/phase-3-randomized-double-blind-study-adjuvant-pembrolizumab-or-without-v940-participants-resectable-stage-ii-iiib-n2-nsclc-not-achieving-pcr-after.

[10] Merck Sharp & Dohme LLC (2026). A Phase 3, Randomized, Double-Blind, Placebo- and Active-Comparator-Controlled Clinical Study of Adjuvant V940 (mRNA-4157) Plus Pembrolizumab Versus Adjuvant Placebo Plus Pembrolizumab in Participants with Resected Stage II, IIIA, IIIB (N2) Non-Small Cell Lung Cancer (INTerpath-002). https://cancer.osu.edu/for-patients-and-caregivers/learn-about-cancers-and-treatments/innovation-at-the-james/clinical-trials/find-a-clinical-trial/a-phase-iii-randomized-doubleblind-placebo.

[11] Sethna Z., Guasp P., Reiche C., Milighetti M., Ceglia N., Patterson E., Lihm J., Payne G., Lyudovyk O., Rojas L.A. (2025). RNA Neoantigen Vaccines Prime Long-Lived CD8+ T Cells in Pancreatic Cancer. Nature.

[12] Huber F., Arnaud M., Stevenson B.J., Michaux J., Benedetti F., Thevenet J., Bobisse S., Chiffelle J., Gehert T., Müller M. (2025). A Comprehensive Proteogenomic Pipeline for Neoantigen Discovery to Advance Personalized Cancer Immunotherapy. Nat. Biotechnol..

[13] Xia H., Hoang M.H., Schmidt E., Kiwala S., McMichael J., Skidmore Z.L., Fisk B., Song J.J., Hundal J., Mooney T. (2024). pVACview: An Interactive Visualization Tool for Efficient Neoantigen Prioritization and Selection. Genome Med..

[14] Imani S., Li X., Chen K., Maghsoudloo M., Jabbarzadeh Kaboli P., Hashemi M., Khoushab S., Li X. (2025). Computational Biology and Artificial Intelligence in mRNA Vaccine Design for Cancer Immunotherapy. Front. Cell. Infect. Microbiol..

[15] Sun S., Liu L., Zhang J., Sun L., Shu W., Yang Z., Yao H., Zhang Z. (2025). The Role of Neoantigens and Tumor Mutational Burden in Cancer Immunotherapy: Advances, Mechanisms, and Perspectives. J. Hematol. Oncol..

[16] Rappaport A.R., Kyi C., Lane M., Hart M.G., Johnson M.L., Henick B.S., Liao C.-Y., Mahipal A., Shergill A., Spira A.I. (2024). A Shared Neoantigen Vaccine Combined with Immune Checkpoint Blockade for Advanced Metastatic Solid Tumors: Phase 1 Trial Interim Results. Nat. Med..

[17] Lopez J., Powles T., Braiteh F., Siu L.L., LoRusso P., Friedman C.F., Balmanoukian A.S., Gordon M., Yachnin J., Rottey S. (2025). Autogene Cevumeran with or without Atezolizumab in Advanced Solid Tumors: A Phase 1 Trial. Nat. Med..

[18] Rojas L.A., Sethna Z., Soares K.C., Olcese C., Pang N., Patterson E., Lihm J., Ceglia N., Guasp P., Chu A. (2023). Personalized RNA Neoantigen Vaccines Stimulate T Cells in Pancreatic Cancer. Nature.

[19] Wu P., Ge J., Hou X., Qu H., Ouyang J., Wang D., Tong T., Meng Y., Yan Q., Shi L. (2026). A circRNA Neoantigen Vaccine Elicits Potent Antitumor Immunity and Synergizes with Checkpoint Blockade in Melanoma. Cancer Lett..

[20] Katsikis P.D., Ishii K.J., Schliehe C. (2024). Challenges in Developing Personalized Neoantigen Cancer Vaccines. Nat. Rev. Immunol..

[21] McCann K., von Witzleben A., Thomas J., Wang C., Wood O., Singh D., Boukas K., Bendjama K., Silvestre N., Nielsen F.C. (2022). Targeting the Tumor Mutanome for Personalized Vaccination in a TMB Low Non-Small Cell Lung Cancer. J. Immunother. Cancer.

[22] Zanotta S., Galati D., De Filippi R., Pinto A. (2024). Enhancing Dendritic Cell Cancer Vaccination: The Synergy of Immune Checkpoint Inhibitors in Combined Therapies. Int. J. Mol. Sci..

[23] Jhunjhunwala S., Hammer C., Delamarre L. (2021). Antigen Presentation in Cancer: Insights into Tumour Immunogenicity and Immune Evasion. Nat. Rev. Cancer.

[24] Roerden M., Castro A.B., Cui Y., Harake N., Kim B., Dye J., Maiorino L., White F.M., Irvine D.J., Litchfield K. (2024). Neoantigen Architectures Define Immunogenicity and Drive Immune Evasion of Tumors with Heterogenous Neoantigen Expression. J. Immunother. Cancer.

[25] Fournier C., Mercey-Ressejac M., Derangère V., Al Kadi A., Rageot D., Charrat C., Leroy A., Vollaire J., Josserand V., Escudé M. (2025). Nanostructured Lipid Carriers Based mRNA Vaccine Leads to a T Cell-Inflamed Tumour Microenvironment Favourable for Improving PD-1/PD-L1 Blocking Therapy and Long-Term Immunity in a Cold Tumour Model. EBioMedicine.

[26] Persico I., Doro M.G., Frogheri L., Sini M.C., Maestrale G.B., Manca A., Mallardo D., Ascierto P.A., Palmieri G. (2026). How Deeply Can mRNA Vaccines Affect the Responsiveness to Immune Checkpoint Inhibitors Through Changes in the Tumor Microenvironment? Evidence from Melanoma. Cells.

[27] Yu X., Fu K. (2026). Neoantigen-Based Cancer Vaccines: A Mechanistic and Clinical Review of Personalised Melanoma Immunotherapy. Front. Immunol..

[28] Zoroddu S., Bagella L. (2025). Next-Generation mRNA Vaccines in Melanoma: Advances in Delivery and Combination Strategies. Cells.

[29] Gainor J.F., Patel M.R., Weber J.S., Gutierrez M., Bauman J.E., Clarke J.M., Julian R., Scott A.J., Geiger J.L., Kirtane K. (2024). T-Cell Responses to Individualized Neoantigen Therapy mRNA-4157 (V940) Alone or in Combination with Pembrolizumab in the Phase 1 KEYNOTE-603 Study. Cancer Discov..

[30] Carlino M.S., Khattak A., Meniawy T., Ansstas G., Taylor M.H., Kim K.B., McKean M., Long G.V., Sullivan R.J., Faries M. (2026). Three-Year Update of a Randomized Phase IIb Study of the Individualized Neoantigen Therapy Intismeran Autogene (mRNA-4157, V940) Plus Pembrolizumab Versus Pembrolizumab in Resected Melanoma. JCO Oncol. Adv..

[31] Merck Sharp & Dohme LLC (2026). A Phase 2, Randomized, Double-Blind, Placebo- and Active-Comparator-Controlled Clinical Study of V940 (mRNA-4157) Plus Pembrolizumab Versus Placebo Plus Pembrolizumab in Participants with First-Line Advanced Melanoma (INTerpath-012). https://www.istitutotumori.mi.it/en/clinical-trials/studio-clinico-fase-2-randomizzato-doppio-cieco-v940-mrna-4157-piu-pembrolizumab-rispetto-placebo-piu-pembrolizumab-prima-linea-partecipanti-melanoma.

[32] Weber J.S., Luke J.J., Carlino M.S., Khattak M.A., Meehan R.S., Brown M., Zhang J., Krepler C., Duic J.P., Long G.V. (2024). INTerpath-001: Pembrolizumab with V940 (mRNA-4157) versus Pembrolizumab with Placebo for Adjuvant Treatment of High-Risk Stage II-IV Melanoma. J. Clin. Oncol..

[33] Merck & Co., Inc., Moderna, Inc. (2026). Merck and Moderna Announce Phase 3 INTerpath-001 Trial of Intismeran Autogene Plus KEYTRUDA^®^ Met Endpoints of Recurrence-Free Survival (RFS) and Distant Metastasis-Free Survival (DMFS) in Patients with Completely Resected Stage IIB–IV Melanoma. https://www.merck.com/news/merck-and-moderna-announce-phase-3-interpath-001-trial-of-intismeran-autogene-plus-keytruda-met-endpoints-of-recurrence-free-survival-rfs-and-distant-metastasis-free-survival-dmfs-in-patient/.

[34] Tang Z., Zha L., Liang R., Li T. (2026). Lung Cancer Vaccines to Enhance Immune Checkpoint Inhibitor Therapy: Evidence and Future Perspectives. J. Hematol. Oncol..

[35] Yu J., Kong X., Feng Y. (2025). Tumor Microenvironment-Driven Resistance to Immunotherapy in Non-Small Cell Lung Cancer: Strategies for Cold-to-Hot Tumor Transformation. Cancer Drug Resist..

[36] Merck Sharp & Dohme LLC (2026). A Phase 2, Randomized, Double-Blind, Placebo-Controlled Study of V940 in Combination with Pembrolizumab and Chemotherapy as First-Line Treatment for Participants with Metastatic Squamous NSCLC (INTerpath-013). https://www.istitutotumori.mi.it/en/clinical-trials/phase-2-randomized-double-blind-placebo-controlled-study-v940-combination-pembrolizumab-and-chemotherapy-first-line-treatment-participants-metastatic.

[37] McGranahan N., Furness A.J.S., Rosenthal R., Ramskov S., Lyngaa R., Saini S.K., Jamal-Hanjani M., Wilson G.A., Birkbak N.J., Hiley C.T. (2016). Clonal Neoantigens Elicit T Cell Immunoreactivity and Sensitivity to Immune Checkpoint Blockade. Science.

[38] Al Bakir M., Reading J.L., Gamble S., Rosenthal R., Uddin I., Rowan A., Przewrocka J., Rogers A., Wong Y.N.S., Bentzen A.K. (2025). Clonal Driver Neoantigen Loss under EGFR TKI and Immune Selection Pressures. Nature.

[39] Genentech, Inc. (2026). A Phase II, Open-Label, Multicenter, Randomized Study of the Efficacy and Safety of Adjuvant Autogene Cevumeran Plus Atezolizumab and mFOLFIRINOX Versus mFOLFIRINOX Alone in Patients with Resected Pancreatic Ductal Adenocarcinoma. https://www.cancer.columbia.edu/cancer-types-care/clinical-trials/find-clinical-trial/phase-ii-open-label-multicenter-randomized-study-efficacy-and-safety-adjuvant-autogene-cevumeran-plus-atezolizumab-and-mfolfirinox-versus-mfolfirinox-alone-patients-resected-pancreatic-ductal.

[40] Balachandran V.P., Łuksza M., Zhao J.N., Makarov V., Moral J.A., Remark R., Herbst B., Askan G., Bhanot U., Senbabaoglu Y. (2017). Identification of Unique Neoantigen Qualities in Long-Term Survivors of Pancreatic Cancer. Nature.

[41] Masugi Y. (2022). The Desmoplastic Stroma of Pancreatic Cancer: Multilayered Levels of Heterogeneity, Clinical Significance, and Therapeutic Opportunities. Cancers.

[42] Palmer C.D., Rappaport A.R., Davis M.J., Hart M.G., Scallan C.D., Hong S.-J., Gitlin L., Kraemer L.D., Kounlavouth S., Yang A. (2022). Individualized, Heterologous Chimpanzee Adenovirus and Self-Amplifying mRNA Neoantigen Vaccine for Advanced Metastatic Solid Tumors: Phase 1 Trial Interim Results. Nat. Med..

[43] Genentech, Inc. (2026). A Phase II, Open-Label, Multicenter, Randomized Study of the Efficacy and Safety of RO7198457 in Combination with Pembrolizumab Versus Pembrolizumab in Patients with Previously Untreated Advanced Melanoma. https://www.dana-farber.org/clinical-trials/19-022.

[44] Braun D.A., Moranzoni G., Chea V., McGregor B.A., Blass E., Tu C.R., Vanasse A.P., Forman C., Forman J., Afeyan A.B. (2025). A Neoantigen Vaccine Generates Antitumour Immunity in Renal Cell Carcinoma. Nature.

[45] Yi M., Zheng X., Niu M., Zhu S., Ge H., Wu K. (2022). Combination Strategies with PD-1/PD-L1 Blockade: Current Advances and Future Directions. Mol. Cancer.

[46] Imani S., Jabbarzadeh Kaboli P., Babaeizad A., Maghsoudloo M. (2025). Neoantigen mRNA Vaccines and A2A Receptor Antagonism: A Strategy to Enhance T Cell Immunity. Hum. Vaccin. Immunother..

